# Comparative Genomics and Phylogenetics of Chloroplasts Reveal Lower Rates of Genetic Variation in Mango (*Mangifera*)

**DOI:** 10.1002/ece3.71957

**Published:** 2025-08-08

**Authors:** Chuanning Li, Jianfeng Huang, Zuan Wei, Yuzu Gao, Gulbar Yisilam, Enting Zheng, Fangfang Jiao, Zhenzhou Chu, Ying Su, Caihua Liao, Xuefen Lei, Jinyu Nong, Yonghai Liang, Juanyan Lin, Yingman Wei, Yu Zhang, Qiuyan Wang, Xinmin Tian

**Affiliations:** ^1^ Key Laboratory of Ecology of Rare and Endangered Species and Environmental Protection (Ministry of Education) & Guangxi Key Laboratory of Landscape Resources Conservation and Sustainable Utilization in Lijiang River Basin College of Life Science, Guangxi Normal University Guilin China; ^2^ Guangxi University Engineering Research Center of Bioinformatics and Genetic Improvement of Specialty Crops, College of Life Science Guangxi Normal University Guilin China; ^3^ National Key Laboratory of Tropical Crop Breeding, Tropical Crops Genetic Resources Institute Chinese Academy of Tropical Agricultural Sciences Haikou China; ^4^ Xinjiang Key Laboratory of Biological Resources and Genetic Engineering, College of Life Science and Technology Xinjiang University Urumqi China; ^5^ Guangxi Subtropical Research Institute Guangxi Academy of Agricultural Sciences Nanning China

**Keywords:** chloroplast genome, comparative genomics, genetic variation, mango, phylogenetic

## Abstract

The mango is an important economic crop with a long history of cultivation. However, studies on the characteristics among chloroplast (cp) genomes and the phylogenetic relationships of different mango varieties are still limited. To fill this research gap, we assembled, annotated, and compared the cp genomes of 23 mango germplasms. The mango cp genome exhibited a typical quadripartite structure, ranging in length from 157,604 to 158,949 bp. Each sequence encoded 129 genes, including 84 protein‐coding genes, 37 tRNA genes, and 8 rRNA genes. Nucleotide diversity analysis identified three mutation hotspot regions, *trn*H‐GUG‐*psb*A, *ycf*4‐*cem*A, and *ndh*F‐*rpl*32, which could be used to develop chloroplast‐specific markers. Phylogenetic analysis revealed that mango germplasm can be divided into four major clades, with wild and cultivated varieties forming independent clades. Interestingly, comparative chloroplast genomics and phylogenetics revealed a relatively low rate of genetic variation among cultivated mangoes. This phenomenon may be attributed to extensive interspecific hybridization and gene introgression events during mango domestication. This study provides valuable genomic resources for crop breeding and enhances our understanding of the genetic variation and phylogenetic relationships among different mango varieties.

## Graphical abstract

Three highly variable sites were identified as chloroplast‐specific markers. Low rate of genetic variation in the mango chloroplast genome. Gene introgression and hybridization affect the mango genetic diversity.

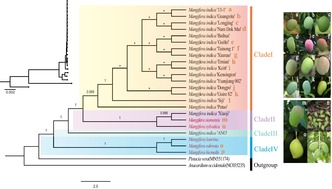

**Abbreviations**
AT: adenine–thymine
BI: Bayesian inference
CDS: protein‐coding sequence
cp: chloroplast
ENC: effective number of codons
GC: guanine–cytosine
IR: inverted repeat region
LSC: large single‐copy region

*M. indica*

:
*Mangifera indica*

Pi: nucleotide diversity
PP: posterior probabilities
RSCU: relative synonymous codon usage
SSC: small single‐copy region
SSRs: simple sequence repeats

## 1 Background

Mango is a significant economic crop in the genus *Mangifera* of the Anacardiaceae family. It is often hailed as the “king of fruits” and is widely cultivated in tropical and subtropical regions (Tharanathan et al. 2006). Mango is known for its distinctive taste and flavor and is rich in vitamins, proteins, carotenoids, and other beneficial phytochemicals (Niu et al. 2022). The genus *Mangifera* includes approximately 69 species, with approximately 15 being edible, and is primarily found in regions such as India, Bangladesh, the Malay Peninsula, and Southeast Asia. Among these species, *M. indica* stands out as the most extensively cultivated mango species worldwide (Chen, Dang, et al. 2020). It is generally accepted that India and Southeast Asia are the two centers of origin of mangoes. Mangos have been cultivated in India for more than 4000 years, and more than 1000 varieties have been cultivated (Mukherjee and Litz 2009). Since the 14th century, mango has spread globally and has been cultivated in China for more than 1300 years (Sawangchote et al. 2009). Five mango varieties have been recorded in the *Flora of China*, that is, *M. siamensis* , *M. hiemalis* , *M. laurina* , *M. sylvatica* , and the widely cultivated *M. indica* (Editorial Committee of Flora of China 1980). Although mangoes have been domesticated for thousands of years, their genomics research lags significantly behind that of traditional fruit trees such as apples and citrus. It was not until 2014 that the first complete chloroplast genome sequence was completed (Azim et al. 2014). In 2020, the complete chloroplast genome sequence of *M. sylvatica* was subsequently reported, and a phylogenetic tree was constructed to reveal the genetic relationships of mango (Zhang et al. 2020). In 2021, plastid‐to‐mitochondrial genome transfer and mutation hotspots were identified in four mango species, which provided rich genetic information for phylogenetic studies and species identification of mango (Niu et al. 2021). To date, complete cp genomes have been published for only 12 species, including *M. indica* . Therefore, there is an urgent need for further research to strengthen the development and application of mango genomic resources.

Taxonomic studies of mango have undergone several stages of development, and the taxonomic system established by Kostermans and Bompard (1993) is widely used to divide mango into two subgenera, namely, *Mangifera* and *Limus*. The subgenus *Mangifera* contains most cultivated varieties, which are further subdivided into *Euantherae, Deciduae*, and *Rawa*, whereas the subgenus *Limus* is represented by wild species such as *M. pentandra* , which has a distinctive leaf vein structure and inflorescence. However, the prevalence of natural hybridization has led to genetic infiltration, such as that of *M. persiciforma*, which combines hybrid characteristics from two subgenera (Kostermans and Bompard 1993). This makes the classification system based on phenotypic traits limited. More crucially, frequent hybridization between species may accelerate the genetic recombination rate, driving convergent evolution in key traits, which makes it difficult to understand the rate of genetic variation in mango varieties. Therefore, it is necessary to study the phylogenetic relationships within mango and evaluate the rate of genetic variation in mango.

Chloroplast genomes in plants are generally circular DNA molecules, 150–160 kb in size, consisting of a large single‐copy region (LSC), a small single‐copy region (SSC), and two inverted repeat regions (IRs; IRa and IRb) (Jansen et al. 2005; Green 2011; Ivanova et al. 2017; Niu et al. 2021). Compared with the nuclear genome, the cp genome has a simpler structure, slower evolutionary rate, and high degree of conservation, making it crucial for plant identification, classification, and phylogenetic analysis (Nguyen et al. 2021; Zheng et al. 2024; Wu et al. 2024). Consequently, the use of cp genomics methods to explore the structural variations and phylogenetic relationships between wild and cultivated mangoes can increase the understanding of the characteristics and evolutionary history of mango genomes.

This study aimed to (Abdullah et al. 2020) comprehensively assemble and annotate the cp genome of mango and conduct comparative analyses of the structural differences and functional characteristics of the genomes of different mango germplasms; (Azim et al. 2014) identify mutation hotspot regions as chloroplast‐specific markers for future phylogenetic analyses of the genus *Mangifera*; (Beier et al. 2017) conduct phylogenetic analyses using protein‐coding region sequences to reveal relationships among mango species; and (Benson 1999) evaluate the rate of genetic variation among different mango germplasms at the cp genome level, thereby providing different insights into the rate of genetic variation in mango. This research will further our understanding of the characteristics of the mango cp genome and provide a genomic foundation for genetic breeding and cultivar enhancement of mango.

## 2 Materials and Methods

### 2.1 Sample Collection and DNA Extraction

In this study, the 23 mango samples used, along with their fundamental information and geographical origins, are listed in Table 1. Prof. Xinmin Tian and A.R. Jianfeng Huang collected and identified all the newly sequenced plants in the National Mango Germplasm Resource Nursery, Guangxi, China. All voucher samples were deposited at the National Mango Germplasm Resource Nursery, Guangxi, China, with voucher sample numbers FSMG230028**–**FSMG230050. Fresh mango leaves were dried via silica gel. The cetyltrimethylammonium bromide method was employed to extract genomic DNA from the leaves, and the integrity of the extracted DNA was subsequently checked via agarose gel electrophoresis.

**TABLE 1:** The material sources of 23 mango germplasms.

Sample number	Germplasm name	Embryony	Germplasm type	Origin	Voucher samples
1	*M. indica* “Nam Dok Mai”	P	Cultivar	Southeast Asia	FSMG230028
2	*M. indica* “Xiamao”	P	Cultivar	China	FSMG230029
3	*M. indica* “Kensington”	P	Cultivar	Australia	FSMG230030
4	*M. indica* “Guifei”	M	Cultivar	China	FSMG230031
5	*M. indica* “Tainong 1”	M	Cultivar	China	FSMG230032
6	*M. siamensis*	P	Wild	Southeast Asia	FSMG230033
7	*M. indica* “Siji”	P	Cultivar	Southeast Asia	FSMG230034
8	*M. indica* “ANO”	P	Cultivar	China	FSMG230035
9	*M. odorata*	P	Wild	Southeast Asia	FSMG230036
10	*M. indica* “Guire 82”	M	Cultivar	China	FSMG230037
11	*M. indica* “Keitt”	M	Cultivar	USA	FSMG230038
12	*M. indica* “Dongpo”	P	Cultivar	China	FSMG230039
13	*M. indica* “Guangxitu”	P	Cultivar	China	FSMG230040
14	*M. indica* “Ernian”	P	Cultivar	China	FSMG230041
15	*M. hiemalis*	M	Wild	China	FSMG230042
16	*M. indica* “Baihua”	P	Cultivar	China	FSMG230043
17	*M. laurina*	P	Wild	China	FSMG230044
18	*M. indica* “Yuanjiang 002”	P	Cultivar	China	FSMG230045
19	*M. sylvatica*	P	Wild	China	FSMG230046
20	*M. indica* “Xiaoji”	P	Cultivar	China	FSMG230047
21	*M. indica* “Putao”	P	Cultivar	China	FSMG230048
22	*M. indica* “Longjing”	P	Cultivar	China	FSMG230049
23	*M. indica* “13‐1”	P	Cultivar	Israel	FSMG230050

Abbreviations: *M*., *Mangifera*; M, monoembryony; P, polyembryony; U, unknown.

### 2.2 Chloroplast Genome Sequencing, Assembly, and Annotation

DNA samples were sequenced on an Illumina HiSeq 2500 platform using paired‐end sequencing to generate 150 bp reads. Quality control was performed using FastQC v0.12.0 (Chen et al. 2018) to obtain high‐quality data, resulting in approximately 10 GB of clean data per sample. The cp genome was assembled de novo using GetOrganelle v1.7 (Jin et al. 2020) with the following parameters: −R 10 −k 21,45,65,85,105,127 −P 1000000 −F embplant_pt. The assembly outcomes were visualized using Bandage v0.8.1 software (Wick et al. 2015). *M. indica* (MN711724) was used as the reference sequence, and the assembled sequences were screened using Geneious Prime v2024.0.5 (Kearse et al. 2012). The cp genome was annotated using CPGAVAS2 (Shi et al. 2019) with the default parameters. The annotation results were then imported into Geneious for manual review and correction of annotation errors, and the start and stop codon positions of protein‐coding genes were determined based on reference sequences. A gene map of the mango cp genome was generated using OGDRAW v1.3.1 software (Greiner et al. 2019). The GC content, genome length, number of genes, and gene type were determined using Geneious Prime v2024.0.5 (Kearse et al. 2012) software.

### 2.3 Repeated Sequence Analysis

Dispersed repetitive sequences in the mango cp genomes were detected using the online software Reputer (https://bibiserv.cebitec.uni‐bielefeld.de/reputer) (Kurtz et al. 2001), with a maximum computed repeat length of ≥ 50 bp and a minimal repeat size of 8. Tandem repeats were identified using the online tool Tandem Repeat Finder (https://tandem.bu.edu/trf/trf.advanced.submit.html) (Benson 1999) with default parameters. Simple sequence repeats (SSRs) were detected using the MISA tool (https://webblast.ipk‐gatersleben.de/misa/) (Beier et al. 2017) with the following thresholds for mono‐, di‐, tri‐, tetra‐, penta‐, and hexa‐nucleotide repeats: 10, 5, 4, 3, 3, and 3, respectively.

### 2.4 Analysis of IR Boundary Contraction and Expansion

The online software CPJSdraw v1.0.0 (Li et al. 2023) was used to compare the variations in the boundary contraction and expansion of the LSC, IRb, SSC, and IRa regions among 23 mango germplasms.

### 2.5 Sequence Variation Analysis

The online software mVISTA (https://genome.lbl.gov/vista/mvista/submit.shtml) (Frazer et al. 2004) was used to analyze the sequence differences between different mango germplasms, with *M. indica* (MN711724) as the reference sequence. Collinearity analysis was conducted using the Mauve plugin in Geneious software (Darling et al. 2004).

### 2.6 Codon Usage Bias Analysis

We used CodonW v1.4.2 (http://codonw.sourceforge.net) to analyze codon usage in the protein‐coding regions of the mango cp genome. Coding sequences (CDSs) longer than 300 bp were selected, and 52 CDS genes per sequence were retained for subsequent analysis. CodonW v1.4.2 software was used to calculate the relative synonymous codon usage, effective number of codons (ENC), and GC content (Liang et al. 2022).

### 2.7 Nucleotide Diversity Analysis

In this study, to compare nucleotide diversity among different mango varieties, we used the MAFFT plugin in Geneious Prime v2024.0.5 to align 23 mango cp genome sequences. We subsequently calculated nucleotide diversity using DnaSP v6.10 (Rozas et al. 2017), with a window length of 600 bp and a step size of 200 bp.

### 2.8 Phylogenetic Analysis

*Pistacia vera* (MN551174) and *Anacardium occidentale* (NC035235) were selected as outgroups. The mango and outgroup sequences were aligned via the default settings of MAFFT v7 (Katoh and Standley 2013), followed by the elimination of ambiguously aligned regions with Gblocks v0.91b (Talavera and Castresana 2007). The replacement models were determined using PartitionFinder2 (Lanfear et al. 2017) based on the Bayesian information criterion. The Bayesian tree was constructed using MrBayes v3.2 (Ronquist et al. 2012), employing the Markov chain Monte Carlo algorithm running for 10,000,000 generations with sampling every 1000 generations. The generated tree files were uploaded to FigTree v1.4.4 (http://tree.bio.ed.ac.uk/software/figtree/) for visualization.

## 3 Results

### 3.1 Structure and Basic Characterization of the Chloroplast Genome

**FIGURE 1:**
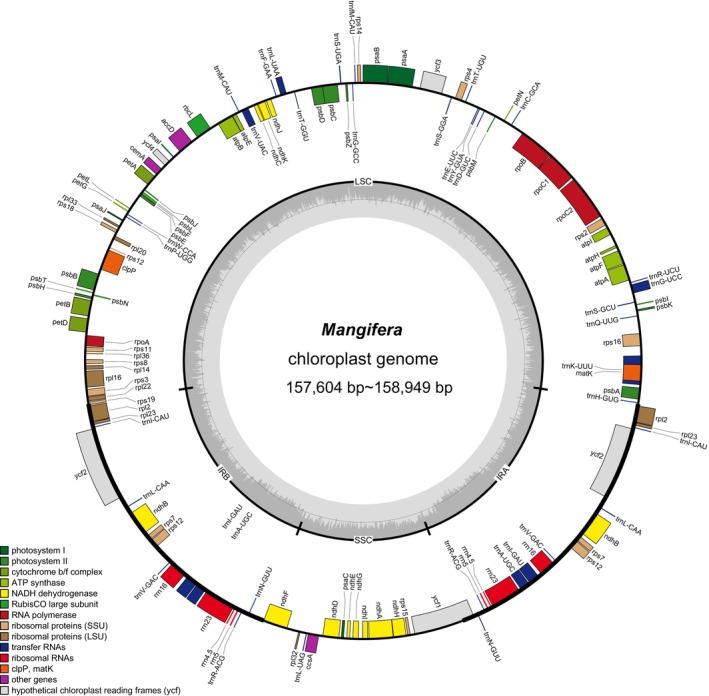
Gene maps of the complete chloroplast genomes of *Mangifera* species. The genes shown on the outside of the outer circle are transcribed counterclockwise, and those on the inside are transcribed clockwise. The inner circle displays the AT and GC makeup of the genome as light gray and dark, respectively. Inverted repeats are designated by the letters IRb and IRa, small single‐copy regions by SSC, and large single‐copy regions by LSC.

In this study, based on consistent gene content, order, and orientation, we used 1 cp gene map to represent all 23 mango cp genomes (Figure 1). The lengths of the cp genomes of the 23 mango germplasms ranged from 157,604 bp ( *M. indica* “ANO”) to 158,949 bp ( *M. laurina* ) (Figure 1; Table S1). All mango cp genomes have a typical quadripartite structure, consistent with known angiosperms, including an LSC region, an SSC region, and two IR regions. The GC content of all mango germplasms was highly conserved, ranging from 37.8% to 37.9%. Specifically, the GC content varied among the different regions: in the LSC region, it ranged from 35.8% to 36.0%; however, in the IR region, it was consistently 43.0%, and in the SSC region, it ranged from 32.3% to 32.4%. Studies have shown that the IR region has a significantly higher GC content than the LSC and SSC regions. Furthermore, 129 genes were annotated in the mango cp genome, including 84 protein‐coding genes, 8 rRNA genes, and 37 tRNA genes (Table S1). Based on gene function, the 129 genes were classified into four categories (Table S2): self‐replication genes, photosynthesis genes, genes with other functions (*mat*K, *cem*A, *ccs*A, *acc*D and *clp*P), and genes with unknown functions (*ycf*1, *ycf*2, *ycf*3 and *ycf*4). Among these genes, 18 genes contained introns, with three genes (*rps*12, *clp*P and *ycf*3) having two introns and the remaining 15 genes having one intron each. Examination of the start and stop codons in the cp genome revealed that most protein‐coding genes used ATG as the start codon, whereas the *rps*19 CDS used GTG as the start codon. All the CDSs were terminated with one of three stop codons, TAA, TAG, or TGA.

### 3.2 Repeat Sequence Analysis

**FIGURE 2:**
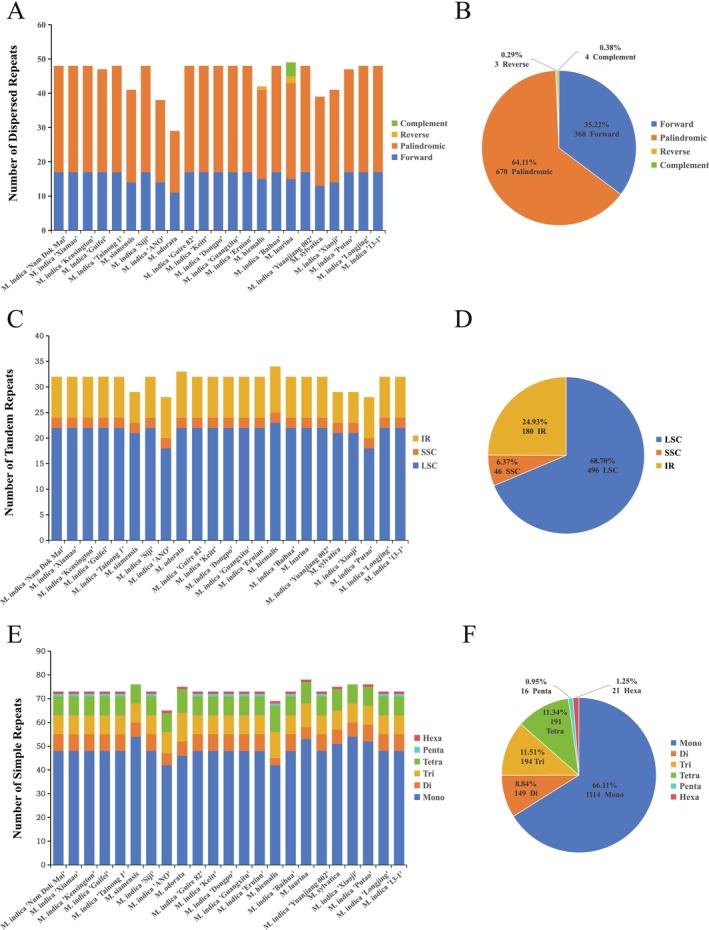
Numbers of three types of repetitive sequences in the chloroplast genomes of 23 mango germplasms. (A) Number of dispersed repeats; (B) Proportion of different dispersed repeat types. (C) Number of tandem repeats in the LSC, SSC, and IR regions, IRs, inverted repeat regions; LSC, large single‐copy region; SSC, small single‐copy region; (D) proportion of tandem repeats in individual regions; (E) number of simple repeats; (F) proportion of different simple repeat types.

Dispersed repetitive sequences in cp genomes are generally of four types: forward repeats (F), reverse repeats (R), palindromic repeats (P), and complementary repeats (C). Among the 23 mango germplasms, the number of dispersed repeats varied from 29 ( *M. odorata* ) to 49 ( *M. laurina* ) (Figure 2A,B). Palindromic repeats were the most abundant, totaling 670 and representing 64.11% of the repetitive sequences; whereas reverse and complementary repeats accounted for only 0.7% (Figure 2B). Complementary repeats are rare in cp genomes, occurring only four times in *M. laurina* . Notably, *M. laurina* is the only mango germplasm among the 23 analyzed that possesses four types of repeats.

A total of 722 tandem repeat sequences were identified. The number of tandem repeats varied among the different germplasms, ranging from 28 ( *M. indica* “ANO” and *M. indica* “Putao”) to 34 ( *M. hiemalis* ) (Figure 2C). Analysis of their distribution revealed that most of these sequences were in the LSC region (496, 68.70%), whereas the SSC region contained the fewest sequences (46, 6.37%) (Figure 2D).

A total of 1685 SSRs were detected across 23 mango germplasms, which were classified into six types (Figure 2E,F). Mononucleotide repeats were the most abundant, totaling 1114 repeats (65.11%). In contrast, pentanucleotide (0.95%) and hexanucleotide (1.25%) repeats were relatively rare. Among the mononucleotide repeats, A/T repeats were predominant (61.78%).

### 3.3 Analysis of IR Boundary Contraction and Expansion

The IR region of the cp genome is relatively conserved. However, boundary gene sequences may expand outward or contract inward (Daniell et al. 2016). The mango cp genome contains four boundaries: LSC/IRb (JLB), IRb/SSC (JSB), SSC/IRa (JSA) and IRa/LSC (JLA) (Figure S1). The *rps*19 gene serves as a boundary gene for the LSC/IRb region, measuring 285 bp, with 81–104 bp extending into the IRb region. The *ndh*F gene spans the IRb/SSC boundary, with a length of 34–36 bp in the IRb region and 2211–2213 bp in the LSC region. In the SSC/IRa region, the *ycf*1 gene measured 5613–5630 bp, with 1099–1101 bp in the IRa region. In addition, the *trn*H gene was positioned on the right side of the IRa/LSC boundary, with 58 bp contracted in the LSC region across all mango germplasms.

### 3.4 Sequence Variation Analysis

**FIGURE 3:**
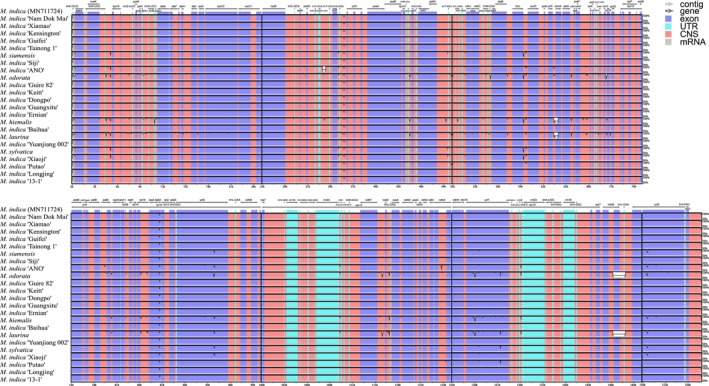
Sequence alignment of mango chloroplast genomes. *M. indica* (MN711724) was used as a reference. The *y*‐axis indicates the percent identity between 50% and 100%. The white peaks indicate the regions with sequence variation among mango germplasms. The purple, blue, pink and gray bars correspond to exons, untranslated regions, noncoding sequences and mRNAs, respectively.

The cp genome sequences of 23 mango germplasms were compared using mVISTA, with *M. indica* (MN711724) serving as the reference (Figure 3). The variations observed in the mango germplasm were predominantly located in noncoding regions, whereas coding regions demonstrated a high degree of conservation. The LSC and SSC regions presented greater variation than did the highly conserved IR regions. Moreover, the number of variant sites in the intergenic regions was significantly greater than that in the genic regions. For example, variations were observed in the *ycf*4‐*cem*A region for *M. hiemalis* and *M. laurina* and in the *ndh*B‐*trn*L‐CAA region for *M. odorata* and *M. laurina* . Collinearity refers to genetic linkage relationships in which homologous genes are arranged in the same order on chromosomes of different species (Tang et al. 2008). The 23 mango cp genomes were analyzed for collinearity. The results revealed that the structure of the mango cp genome remained stable; no rearrangement or inversion was detected, and the gene arrangements were essentially consistent (Figure S2).

### 3.5 Codon Usage Bias Analysis

**FIGURE 4:**
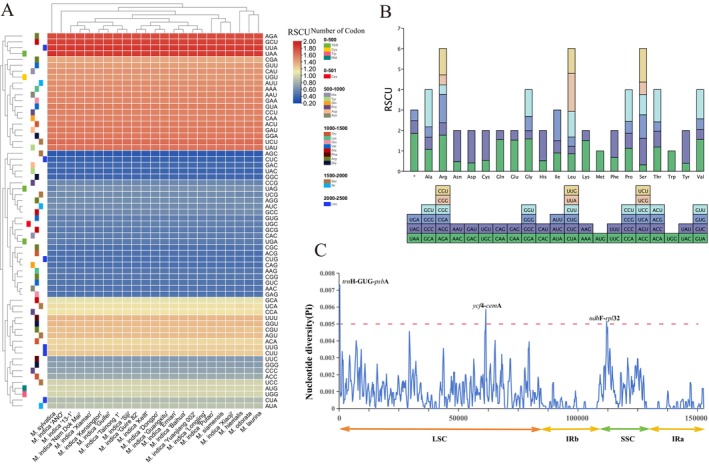
Comparative analysis of codon usage bias and nucleotide diversity in mango chloroplast genomes. (A) Heatmap of RSCU values among 23 mango germplasms. The RSCU value increases as the red color becomes darker. The RSCU value decreases as the blue color darkens. The various codons are represented by each row. Each different species of mango is represented by a different column. (B) Codon content of 21 amino acids and stop codons in chloroplast genes of mango ( *M. hiemalis* as an example). The histogram on the left‐hand side of each amino acid denotes codon usage within mango chloroplast genomes, and the right‐hand side denotes the codon RSCU values. The colors correspond to the codons listed underneath the columns. (C) Nucleotide diversity analysis of mango germplasm. Window length: 600 bp; step size: 200 bp. *x*‐axis: Range of sequence length; *y*‐axis: Pi value.

The RSCU is a metric used to quantify and characterize codon usage bias. When RSCU = 1, there is no preference for synonymous codons; RSCU > 1 indicates a strong preference, whereas RSCU < 1 suggests a weak preference. Among the 23 mango germplasms (Figure 4A,B), 30 codons had RSCU values higher than 1. Among these, 29 codons ended with A or U, whereas only one ended with G (UUG, RSCU = 1.22). These findings suggest that the codon preference of the mango cp genome is associated with A/T. Conversely, 32 codons had RSCU values lower than 1, with AGC having the lowest value of 0.32. There were no significant preferences for the AUG and UGG codons. The effective number of codons (ENC) quantifies the extent to which there is a preference for the nonequilibrium use of synonymous codons, with a theoretical range from 20 to 61. For the 23 mango germplasms, the ENC values ranged from 50.02 to 50.08 (Table S3).

### 3.6 Nucleotide Diversity Analysis

To assess the degree of sequence variation within the mango cp genome, nucleotide diversity (Pi) values for the 23 mango germplasm accessions were calculated using DnaSP v6.10 (Rozas et al. 2017) (Figure 4C). Three mutation hotspot regions were identified: *trn*H‐GUG‐*psb*A, *ycf*4‐*cem*A, and *ndh*F‐*rpl*32 (Pi > 0.005). Of these, *ycf*4‐*cem*A displayed the highest level of variability (Pi = 0.0073). The Pi values varied between 0 and 0.0073, with the lowest values in the IR region, indicating that the IR region of the mango cp genome was conserved and experienced a relatively low evolutionary rate.

### 3.7 Phylogenetic Analysis

**FIGURE 5:**
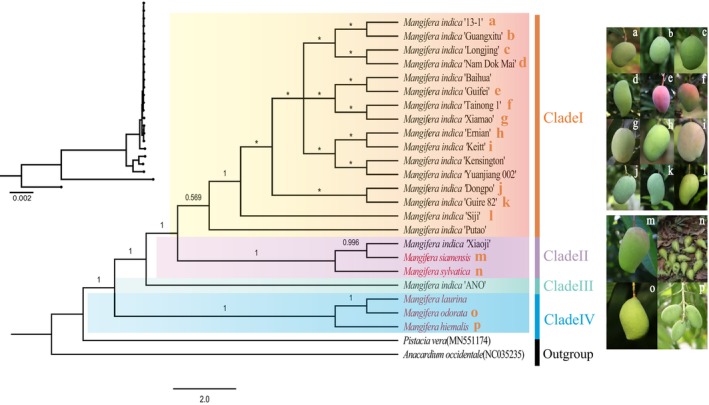
A phylogenetic tree of 23 mango germplasm accessions was constructed using protein‐coding sequences via Bayesian inference. The numbers above the branches represent posterior probabilities (PP). An asterisk (*) denotes a posterior probability of less than 0.50. The cladogram in the upper–left corner illustrates the branch lengths of the phylogenetic tree. Varieties marked in red are wild types. Images a–p on the right correspond to labels a–p on the phylogenetic tree, with a–l representing cultivars and m–p denoting wild species. Image “p” is from the China Plant Image Bank; image “n” is from https://powo.science.kew.org/taxon/urn:lsid:ipni.org:names: 978619‐1; the rest of the images are provided by the National Mango Germplasm Resource Nursery, Guangxi, China.

Using *Pistacia vera* and *Anacardium occidentale* as outgroups, a Bayesian inference (BI) tree was constructed using 77 protein‐coding genes (Figure 5) and whole‐genome sequences (Figure S3). The results show that the phylogenetic trees constructed by the two methods have similar topologies. The phylogenetic tree was separated into four clades. Clade I consists of cultivated mangoes, and these varieties are evolutionarily closely related and may have a common cultivated ancestor. Clade II includes two wild mangoes ( *M. siamensis* and *M. sylvatica* ) clustered with *M. indica* “Xiaoji”. *M. indica* “ANO” clustered as a separate branch (Clade III). *M. laueana*, *M. hiemalis* , and *M. odorata* clustered together in a highly supported clade IV (PP = 1), and these three wild breeds presented large genetic differences from the other clades.

## 4 Discussion

Among the 23 mango cp genomes, *M. indica* “ANO” presented the smallest cp genome (157,604 bp) and LSC region (86,507 bp), whereas *M. laurina* presented the largest cp genome (158,949 bp) and LSC region (87,773 bp). The length of the cp genome is positively correlated with the length of the LSC region (Huang et al. 2022; Duan et al. 2022). The GC contents of all the mango germplasms were similar. However, there was a significant difference in the GC content among the four regions, reflecting the diversity in the structural and functional requirements of each region (Gao et al. 2024). Although the basic characteristics of mango chloroplasts in this study are consistent with other reported mango cp genomic characteristics, there exist differences in the number of annotated genes. There is a total of 113 genes in the mango cp genome reported by Niu et al. (2021), and Tang et al. (2025) reported 134 genes in *M. indica* . In addition, 18 genes contain introns that play different roles in biological processes. For example, *clp*P is associated with the stress response. These introns are involved in various cellular functions, including processes such as ribosomal protein and fatty acid synthesis, which are essential for cellular growth and development. Despite the emergence of diverse cultivated varieties during mango domestication (Wang et al. 2020), the findings suggest that the mango cp genome remains highly conserved in terms of its length, structure, gene count, type, and GC content, suggesting a low genetic mutation rate in the mango cp genome. The low mutation rate observed in the mango cp genome supports the widely held view that plant chloroplasts evolve slowly, which has implications for understanding the genetic stability of mango cultivars.

Simple sequence repeats (SSRs) are important molecular markers used to investigate genetic diversity, population structure, and evolutionary relationships (Ebert and Peakall 2009; George et al. 2015). A total of 1685 SSRs were detected across the 23 mango germplasms, with A/T mononucleotide repeat sequences being the most abundant. This is because chloroplast SSRs are typically composed of polyA or polyT repeat sequences and seldom include polyG or polyC repeat sequences (Kuang et al. 2011; Ravishankar et al. 2015; Liang et al. 2024). The newly identified SSRs produced in the present study considerably enriched the mango SSR database, facilitating the assessment of genetic diversity, germplasm identification, and molecular marker‐assisted breeding in mangoes.

The contraction and expansion of the IR region is a common evolutionary phenomenon that may lead to changes in the length of the cp genome (Liu et al. 2017; Zhou et al. 2018). IR boundary analysis revealed that the boundary genes of most mango germplasms were highly consistent in type and location. However, some cultivated varieties, such as *M. siamensis* , *M. indica* “ANO,” and *M. hiemalis* , have IR length differences, which may be related to the specific selection pressures on these varieties during the evolutionary process. The positions and lengths of the *rps*19, *ndh*F, *ycf*1, *trn*H, *rpl*2, and *trn*N genes near the IR/SC boundary were basically the same in most varieties. This conservation suggests that these genes play crucial roles in chloroplast function and structure. For instance, *rps*19 is involved in ribosomal protein synthesis, while *ndh*F is associated with the electron transport chain (Li et al. 2024).

The variations in the cp genomes were mainly concentrated in single‐copy regions, whereas the IR regions were highly similar. These results are consistent with those observed in the cp genomes of most angiosperms (Zhang et al. 2016). Notably, sequence variation analysis revealed that the *ycf*4‐*cem*A and *ndh*B‐*trn*L‐CAA regions presented relatively high degrees of variation. Codon usage bias is an important aspect of genome evolution and is closely related to the phylogeny of different species (Ellegren 2004; Wang et al. 2023). Among all mango germplasms, codons encoding Leu were the most abundant, whereas those encoding Cys were the least common. The degeneracy of the genetic code allows for multiple codons to encode a single amino acid, which results in variations in codon usage across different species (Paul et al. 2018; Hong et al. 2020; Sheng et al. 2021). Codon usage bias is due to differences in tRNA abundance across different codons (Chen, Wu, and Deng 2020). Codons with RSCU > 1 typically ended in A/U bases, whereas those with RSCU < 1 mostly ended in G/C bases. The preference for A or U in termination codons within plant cp genomes can be attributed to the high A/T content in these genomes, as well as the influence of mutation pressure and natural selection. This leads to a bias in the use of the end codon (Abdullah et al. 2020; Zhang et al. 2023). The selection of synonymous codons is not random, and analyzing codon preferences plays a key role in understanding species adaptation and the process of molecular evolution (Qin et al. 2024).

Pi quantifies the variation in the nucleic acid sequence within a species, with regions of high variability often acting as molecular markers for studies in phylogenetics and biogeography (Li et al. 2018; Abdullah et al. 2020; Qin et al. 2024). Three mutation hotspot regions were identified in the mango cp genome: *trn*H‐GUG‐*psb*A, *ycf*4‐*cem*A, and *ndh*F‐*rpl*32 (Pi > 0.005). In contrast, the sequences of the IR region demonstrated high conservation, which is consistent with the findings from the mVISTA analysis. Additionally, compared with the study of Xin et al. (2023), this study identified a new highly variable region, *trn*H‐GUG‐*psb*A. This variation could be attributed to the different mango varieties used in the two studies, resulting in differences in nucleotide diversity at the same position.

The cp genome contains genetic data that can be utilized to deduce evolutionary and phylogenetic relationships (Firetti et al. 2017). In phylogenetic trees, wild and cultivated varieties form distinct clades. In Clade I, some cultivated germplasms did not exhibit significant differentiation at the cp genome level. The shorter branch lengths and lower support values indicate that the mango germplasms within this clade have relatively smaller genetic differences. A comparative genome analysis of the cp genomes of different mango varieties revealed that wild mango accessions exhibited higher levels of genetic variation in their cp genomes compared to cultivated varieties. The cp genome of wild species included more types of repeats and a higher number of variant sites, such as *M. odorata* , *M. hiemalis* , and *M. laurina* , with more variations observed in the gene spacer. Whereas the cp genomes of cultivated germplasms presented a high degree of conservation in terms of gene sequence, orientation, and genome length. This genome stability led to an extremely low mutation rate in the cp genome of mango. This phenomenon may be caused by gene infiltration and hybridization (Ma et al. 2024).

In the genus *Mangifera*, closely related varieties show a high degree of interspecific hybridization. This is because mangoes are self‐incompatible and prone to cross‐pollination in natural environments. Therefore, the likelihood of interspecific hybridization increases significantly when these outcrossing species are planted in adjacent areas, which can also be observed in some perennial crops (Feng et al. 2020; Miao et al. 2022; Xia et al. 2023). A population genomics study on mangoes indicated that the domestication process is highly complex and may involve multiple domestication events and interspecific hybridization (Warschefsky and von Wettberg 2019). Specifically, extensive hybridization has led to frequent gene flow between various varieties, resulting in a mixture of genetic information. Furthermore, given the widespread cultivation of *M. indica* , this species is likely to have undergone a certain degree of gene introgression with other plants of the same genus. For example, *M. odorata* has been suggested to have a hybrid origin, which was later verified as a cross between *M. indica* and *M. foetida* (Teo et al. 2002; Natalie et al. 2013), suggesting that there may be gene flow between wild species and *M. indica* .

During the cultivation and selection of mangoes, breeders tend to select individuals with desirable traits, resulting in relatively small genetic differences among cultivated varieties. A typical example is the polyembryonic mango, which is favored for its thin, juicy skin and relatively high resistance to storage and transport. However, polyembryony is thought to be governed by a single dominant gene, which restricts hybridization and recombination, leading to the reproduction of genetically identical individuals and thereby impairing the genetic diversification of the population. Consequently, polyembryony may create a genetic bottleneck that further limits genetic variation in mangoes (Wilkinson et al. 2022).

In view of the above limitations, future studies should expand the sample quantity of genomic studies to explore the mechanism of gene infiltration between wild and cultivated mangoes, as well as the interaction between human selection pressure and natural inheritance. These studies help us understand the genetic diversity of mango more comprehensively and provide a solid scientific basis for mango breeding and genetic resource conservation.

## 5 Conclusion

In this study, comparative and phylogenetic analyses of the cp genomes of wild and cultivated mangoes reveal a low rate of genetic variation in cultivated mangoes. This could be due to mango having undergone extensive gene introgression and interspecific hybridization events during domestication, further illustrating the complexity of its domestication history. Three mutation hotspot regions were identified in the LSC and SSC regions, which may be used to develop chloroplast‐specific markers for distinguishing mango germplasms. Furthermore, phylogenetic analysis revealed that wild mango and cultivated mango formed independent branches with high support rates, suggesting that wild mango and cultivated mango may have maintained different genetic characteristics during evolution. This study enriches the cp genome dataset for mangoes, thereby deepening our understanding of their internal structure and phylogenetic relationships. Future research should integrate cp genome data for more mango species, combined with multiomics datasets such as nuclear and mitochondrial genomes, to further refine the analysis of mango genetic diversity. These findings provide a theoretical foundation for studies on mango germplasm identification, genetic improvement, and breeding applications.

## Author Contributions

**Chuanning Li:** data curation (equal), formal analysis (equal), investigation (equal), methodology (equal), validation (equal), visualization (equal), writing – original draft (equal), writing – review and editing (equal). **Jianfeng Huang:** data curation (equal), investigation (equal), methodology (equal), project administration (equal), supervision (equal), writing – review and editing (equal). **Zuan Wei:** formal analysis (equal), investigation (equal), methodology (equal), validation (equal). **Yuzu Gao:** investigation (equal), visualization (equal). **Gulbar Yisilam:** investigation (equal), methodology (equal), supervision (equal), validation (equal), writing – review and editing (equal). **Enting Zheng:** investigation (equal), methodology (equal). **Fangfang Jiao:** writing – review and editing (equal). **Zhenzhou Chu:** methodology (equal), software (equal). **Ying Su:** methodology (equal), resources (equal). **Caihua Liao:** methodology (equal). **Xuefen Lei:** investigation (equal). **Jinyu Nong:** methodology (equal). **Yonghai Liang:** methodology (equal). **Juanyan Lin:** methodology (equal). **Yingman Wei:** methodology (equal). **Yu Zhang:** investigation (equal), resources (equal), supervision (equal). **Qiuyan Wang:** funding acquisition (equal), investigation (equal), project administration (equal), resources (equal), supervision (equal). **Xinmin Tian:** data curation (equal), funding acquisition (equal), investigation (equal), methodology (equal), project administration (equal), supervision (equal), validation (equal), writing – review and editing (equal).

## Ethics Statement

The authors have nothing to report.

## Consent

The authors have nothing to report.

## Conflicts of Interest

The authors declare no conflicts of interest.

## Supporting information


**Figure S1:** Contraction and expansion diagram of the IR region in the chloroplast genomes.

**Figure S2:** Collinearity analysis diagram of 23 mango germplasms.

**Figure S3:** A phylogenetic tree of 23 mango germplasm accessions was constructed using whole‐genome sequences via Bayesian inference.

**Table S1:** Chloroplast genome length, GC content, and gene number of 23 mango germplasms.

**Table S2:** Classification of mango chloroplast genome annotation genes.

**Table S3:** Effective number of codons (ENCs) in 23 mango germplasms.

## Data Availability

The data presented in the study are deposited in the National Center for Biotechnology Information (NCBI, https://www.ncbi.nlm.nih.gov/) under accession numbers PV019373–PV019395. The data are provided within the manuscript or Supporting Information files.
