# Supplementary material for: Comparative Genomics and Phylogenetics of Chloroplasts Reveal Lower Rates of Genetic Variation in Mango (Mangifera)
Source: Ecol Evol. 2025 Aug 8;15(8):e71957. doi: 10.1002/ece3.71957 (PMC12332423; doi:10.1002/ece3.71957)
Supplement: Supplementary file 1 — Figure S1: Contraction and expansion diagram of the IR region in the chloroplast genomes. [file ECE3-15-e71957-s002.docx]

**Figure S1**


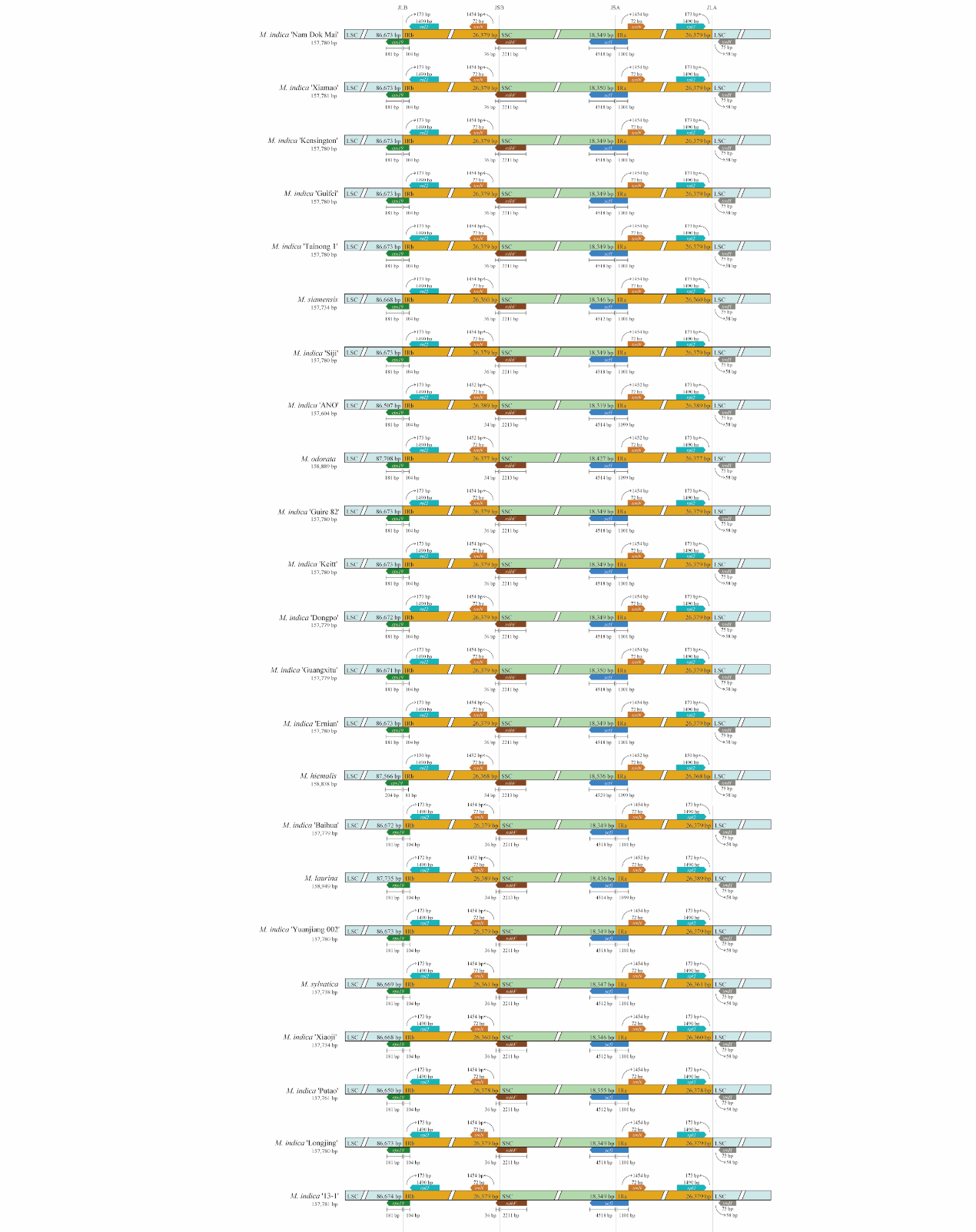


**Fig. S1.** **Contraction and expansion diagram of the IR region in the chloroplast genomes.** The positions of LSC, IR, and SSC junctions were compared among 23 mango germplasm. JLB stands for the junction between the long single copy and reverse repeat (LSC/IRb), JSB stands for the junction between reverse repeat and short single copy (IRb/SSC), JSA stands for the junction between short single copy and forward repeat (SSC/IRa), and JLA stands for the junction between forward repeat and long single copy (IRa/LSC).
