# Supplementary material for: Comparative Genomics and Phylogenetics of Chloroplasts Reveal Lower Rates of Genetic Variation in Mango (Mangifera)
Source: Ecol Evol. 2025 Aug 8;15(8):e71957. doi: 10.1002/ece3.71957 (PMC12332423; doi:10.1002/ece3.71957)
Supplement: Supplementary file 2 — Figure S2: Collinearity analysis diagram of 23 mango germplasms. [file ECE3-15-e71957-s004.docx]

**Figure S2**


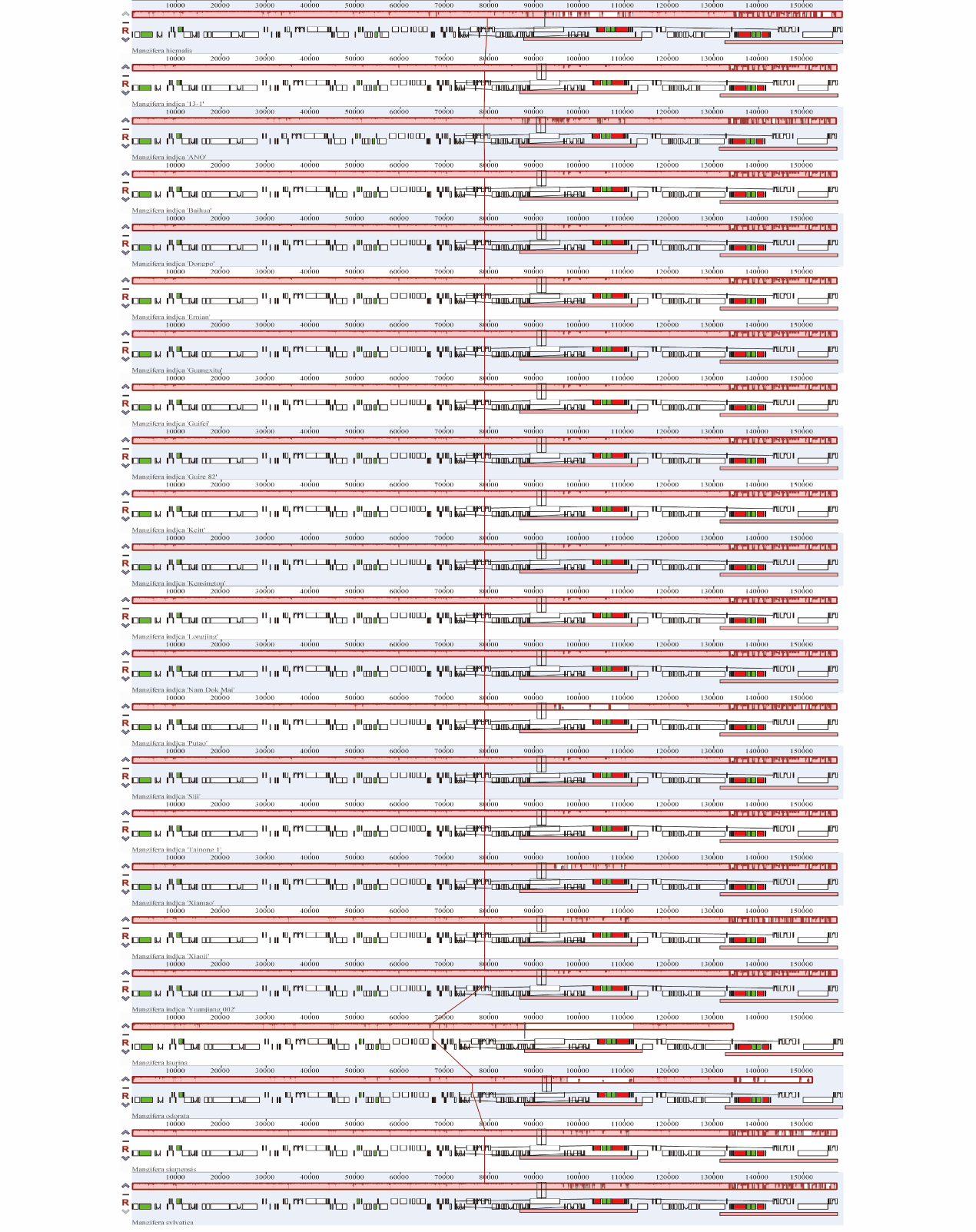


**Fig. S2. Collinearity analysis diagram of 23 mango germplasms.** In this diagram, the long squares depict genomic similarity, and the connecting lines indicate a covariate association. The short squares represent gene locations within each genome. CDS is represented by white squares, tRNA by green squares, and rRNA by red squares.
