# Supplementary material for: Comparative Genomics and Phylogenetics of Chloroplasts Reveal Lower Rates of Genetic Variation in Mango (Mangifera)
Source: Ecol Evol. 2025 Aug 8;15(8):e71957. doi: 10.1002/ece3.71957 (PMC12332423; doi:10.1002/ece3.71957)
Supplement: Supplementary file 3 — Figure S3: A phylogenetic tree of 23 mango germplasm accessions was constructed using whole‐genome sequences via Bayesian inference. [file ECE3-15-e71957-s003.docx]

**Figure S3**


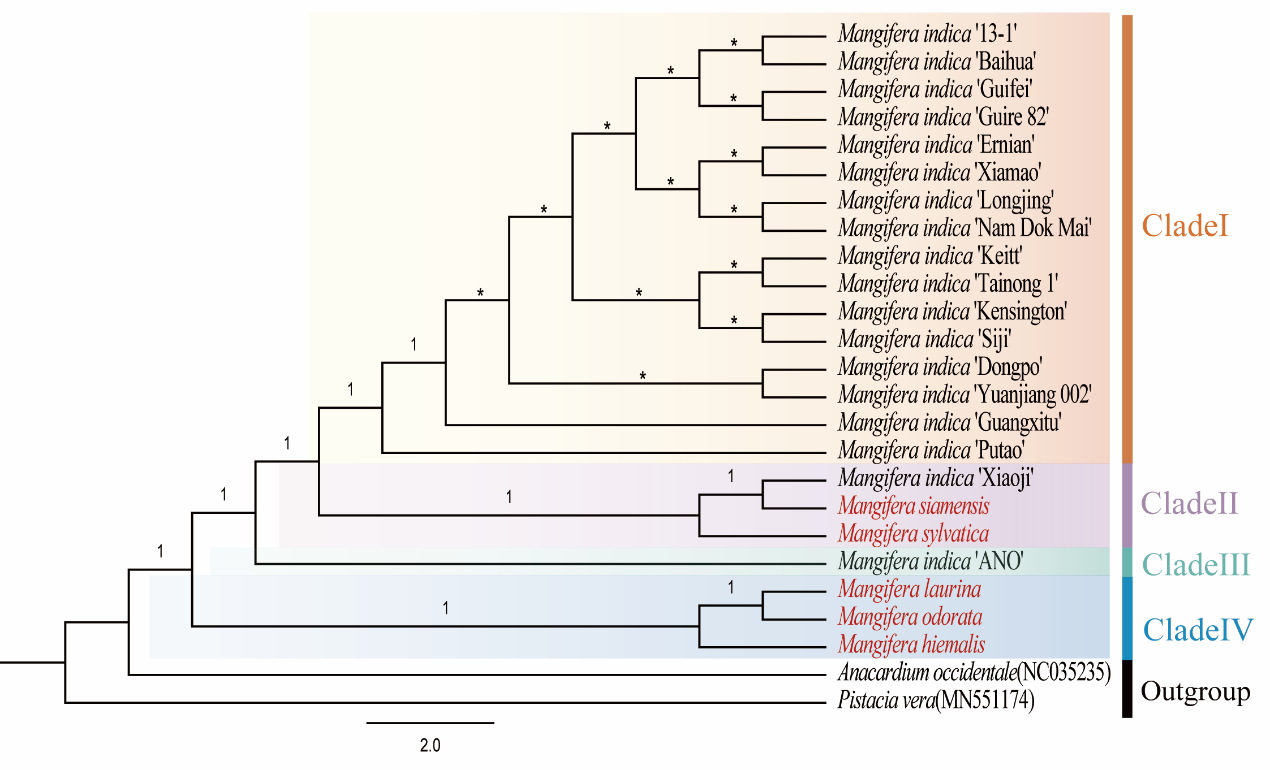


**Fig. S3.** **A phylogenetic tree of 23 mango germplasm accessions was constructed using whole-genome sequences via Bayesian inference.** The numbers above the branches represent posterior probabilities (PP). An asterisk (*) denotes a posterior probability of less than 0.50. Varieties marked in red are wild types.
